# Acoustic Analysis of Apraxia of Speech in a Japanese Patient With Nonfluent/Agrammatic Variant Primary Progressive Aphasia: A Case Study

**DOI:** 10.7759/cureus.90325

**Published:** 2025-08-17

**Authors:** Narihiro Kodama, Akiko Miyazaki, Yuhei Kodani, Ryo Hatazoe, Kosei Hashimoto

**Affiliations:** 1 Department of Speech - Language Pathology and Audiology, Faculty of Rehabilitation, Kawasaki University of Medical Welfare, Kurashiki, JPN; 2 Rehabilitation Center, Kawasaki Medical School Hospital, Kurashiki, JPN; 3 Department of Rehabilitation, Kumamoto Health Science University, Kumamoto, JPN; 4 Department of Speech, Language and Hearing Therapy, Faculty of Health Sciences, Mejiro University, Saitama, JPN

**Keywords:** acoustic analysis, apraxia of speech (aos), nonfluent/agrammatic variant primary progressive aphasia (nfvppa), triangular vowel space area (tvsa), voice onset time (vot)

## Abstract

Apraxia of speech (AOS) is a motor speech disorder often observed in the nonfluent/agrammatic variant of primary progressive aphasia (nfvPPA). While acoustic analysis has proven useful in identifying AOS features in English speakers, data on Japanese speakers are limited. This study investigated the acoustic characteristics of AOS in a 70-year-old Japanese woman diagnosed with corticobasal syndrome and nfvPPA. Speech and neuropsychological assessments revealed typical AOS features. Acoustic measures included triangular vowel space area (tVSA), voice onset time (VOT), and speech duration. As a result, although tVSA was relatively preserved, both VOT and speech duration were prolonged. These findings suggest that VOT and speech duration may serve as effective acoustic markers for detecting AOS in Japanese, providing useful objective indicators for diagnosis and treatment planning.

## Introduction

Apraxia of speech (AOS) is a motor speech disorder characterized by impairments in the programming of speech movements, distinguishing it from aphasia and dysarthria [1]. The main characteristics of AOS include distorted sounds (along with similarly distorted replacement sounds). Speech may also become longer, speech may be noticeably slower, or there may be longer pauses between sounds, syllables, or words. Abnormalities occur in the rhythm and melody (prosody) of the voice due to the sound elongating or stopping [2]. In the left frontal lobe, Broca’s area and the supplementary motor area (SMA) are considered key components of the cortical speech and language network [1]. More recently, in studies of the functional correlates of AOS, the left premotor cortex and precentral gyrus have been identified as primary regions involved, particularly in cases of isolated AOS or when examined using functional MRI or lesion-symptom mapping [3]. In addition, primary progressive aphasia (PPA), a neurodegenerative disorder that progresses slowly without accompanying cognitive or behavioral abnormalities, was first identified by Mesulam [4]. PPA is further divided into subtypes, including the non-fluent/agrammatic variant PPA (nfvPPA), logopenic variant PPA (lvPPA), and semantic variant PPA (svPPA). Among these, nfvPPA is characterized by agrammatism, AOS, or both. While AOS is frequently observed in nfvPPA, it can also occur independently without accompanying aphasia, in which case it is referred to as primary progressive AOS (PPAOS) [5]. Although nfvPPA and PPAOS are clinically distinct syndromes, they share many similarities in their underlying pathology and neuroimaging features. Both are frequently associated with four-repeat tauopathies (4R-tauopathies), such as progressive supranuclear palsy (PSP) and corticobasal degeneration (CBD) [6].

Recent studies have demonstrated that nfvPPA can be distinguished from other PPA variants, such as lvPPA, through detailed acoustic analyses.

For instance, Ballard et al. [7] showed that relative vowel duration during multisyllabic word repetition tasks served as a highly sensitive acoustic marker of AOS and effectively differentiated nfvPPA from lvPPA. Similarly, Haley et al. [8] identified prosodic and articulatory timing abnormalities in connected speech that reliably separated nfvPPA from lvPPA, with narrative speech metrics demonstrating particularly high diagnostic utility. In addition, the utility of acoustic analysis has been reported not only for nfvPPA but also for PPAOS [9].

Research on the acoustic characteristics of AOS in nfvPPA and PPAOS has been increasing in English and other European languages; however, studies involving Japanese speakers remain limited. In particular, there are significant acoustic differences between Japanese and English. For example, while English and German are stress-accent languages, Japanese is a pitch-accent language, making it difficult to directly apply the same acoustic measures used in studies of English speakers to Japanese.

At our institution, we have conducted acoustic analyses of AOS in native Japanese speakers, focusing on voice onset time (VOT), the coefficient of variation of VOT (VOT-CoV), the triangular vowel space area (tVSA), and speech duration. We have previously reported on the acoustic features of PPAOS [10]. However, our past findings were based on a single case, and there is a need to accumulate more cases. It should be noted that the patient presented in the current study is different from the one described in our previous report [10].

The purpose of the present study was to clarify the acoustic characteristics of AOS in the Japanese language by analyzing the speech of a native Japanese speaker diagnosed with nfvPPA. Through this comparison, we sought to evaluate the applicability and effectiveness of acoustic analysis for identifying AOS features in the context of the Japanese language.

The methodology of this study was presented orally at the 18th Annual Meeting of the Japanese Society for Higher Brain Dysfunction, held on November 8, 2024.

## Case presentation

Patient characteristics

**Figure 1:**
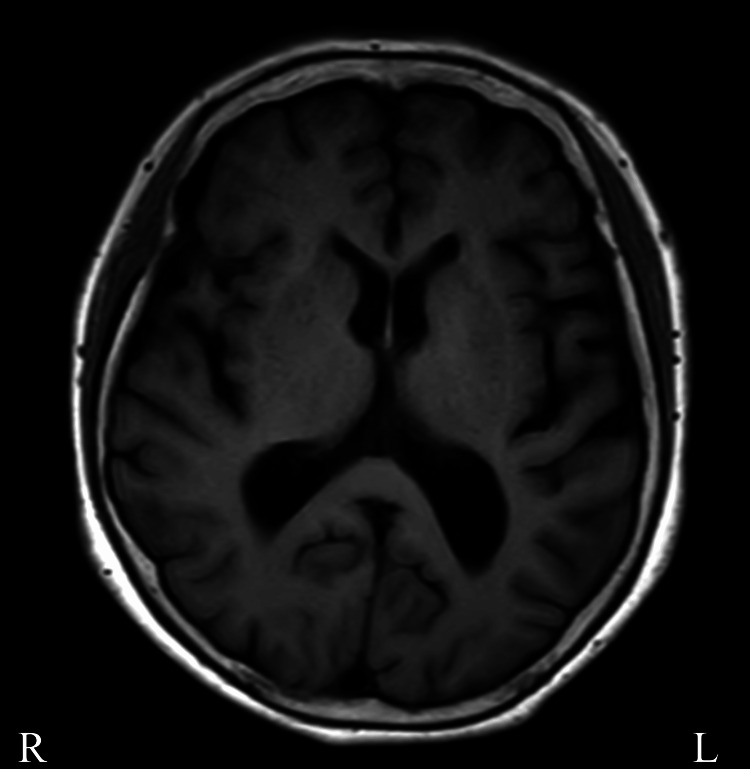
MRI findings Fluid attenuated inversion recovery (FLAIR) image demonstrating prominent atrophy in the left frontal and parietal lobes, with widening of the sulci and ventricular enlargement.

**Figure 2:**
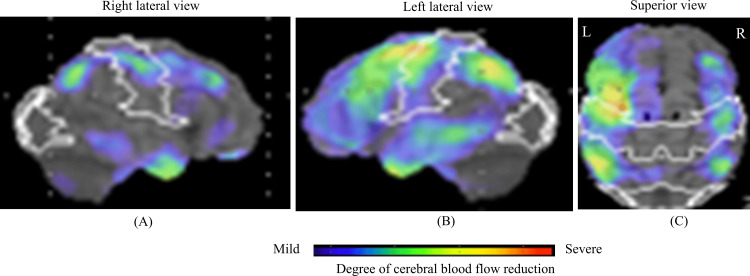
Single-photon emission computed tomography (SPECT) findings Cerebral blood flow (CBF) reduction visualized from three perspectives using 3D surface projections. The color scale indicates the degree of CBF reduction, ranging from mild (blue) to severe (red). (A) Right lateral view showing mild-to-moderate hypoperfusion in the right frontal and parietal cortices. (B) Left lateral view demonstrating more extensive hypoperfusion in the left frontal, temporal, and parietal regions. (C) Superior view highlighting bilateral but asymmetric hypoperfusion, predominantly in the left hemisphere.

The participant was a 70-year-old, right-handed woman who had noticed a gradual slowing of her speech over the past two years. Initially, she visited a local neurosurgical clinic and underwent a brain MRI, which revealed no abnormalities. Subsequently, she reported progressive difficulty in moving her right upper limb and a gradual slowing in her handwriting. Ankyloglossia was noted in her medical history. Two years after symptom onset, she visited our department for further evaluation. Brain imaging was performed, and MRI using fluid attenuated inversion recovery (FLAIR) sequences showed no evidence of acute cerebral infarction or hemorrhage; however, atrophy was observed predominantly in the frontal, parietal, and temporal lobes, with left-sided predominance (Figure 1). Based on these findings, she was diagnosed with corticobasal syndrome (CBS) according to the Armstrong criteria. Additionally, single-photon emission computed tomography (SPECT) imaging revealed decreased cerebral blood flow in the bilateral frontal, parietal, and temporal lobes, with more pronounced hypoperfusion in the left hemisphere (Figure 2). At two years and 10 months post onset, a battery of speech and language assessments was conducted, including Raven’s colored progressive matrices (RCPM), the standard language test of aphasia (SLTA), the frontal assessment battery (FAB), oral function assessment using the supplementary tests for SLTA (SLTA-ST), and voice recording.

Assessment (neurological findings, neuropsychological findings, and oral function evaluation)

The initial neurological examination revealed that the patient was alert and showed no signs of paralysis or sensory impairment; however, difficulty in moving the right upper limb was noted. The patient was independent in both activities of daily living (ADLs) and instrumental ADLs (IADLs).

The results of neuropsychological and oral function assessments were as follows. On the RCPM, the patient scored 29 out of 36, while the FAB yielded a score of 12 out of 18. Language function was evaluated using the SLTA (Japan Society for Higher Brain Dysfunction Brain Function Test Committee, 2003), which is one of the most commonly used standardized assessments for aphasia in Japan. In the word listing task (animal naming), the patient achieved approximately 50% accuracy (eight items). For the tasks of explaining cartoons for "speaking," sentence repetition, and explaining cartoons for "writing," the accuracy was around 80%, while the accuracy for the remaining tasks exceeded 90%. In daily conversational contexts, circumlocution, phonological paraphasia, and grammatical errors (e.g., omission of articles) were observed.

In the oral function assessment, the patient was able to perform tongue protrusion and retraction, lateral tongue movements, exhalation, lip protrusion, throat clearing, forehead wrinkling, and cheek puffing. However, the patient was unable to whistle and stated that they had never been able to do so. When prompted to produce a clicking sound with the tongue, the patient instead protruded the lips, indicating the presence of orofacial apraxia.

Regarding speech characteristics, three speech-language pathologists with clinical experience evaluating 10 or more AOS patients conducted auditory-perceptual evaluations focusing on sound distortions, speech segmentation, sound prolongation, reduced speech rate, and abnormalities in stress and intonation. The speech samples included recordings from the present case, as well as samples from other individuals, and the evaluators were blinded to the identity of the samples. A speech characteristic was judged as abnormal if two or more evaluators identified it as such. As a result, the present case exhibited sound distortions, speech segmentation, reduced speech rate, and abnormal stress and intonation patterns, leading to a diagnosis of apraxia of speech. The respiratory rate was 20 breaths per minute, the maximum sustained expiration time was 7.7 seconds, and the maximum phonation time was 11.5 seconds. Additionally, soft palate elevation during vowel /a/ phonation was adequate, and no nasal emission was observed during phonation when assessed using a nasal mirror. The patient was diagnosed with nfvPPA based on the diagnostic criteria proposed by Gorno-Tempini et al. [11], due to the presence of apraxia of speech and grammatical errors.

Voice recording

Voice recordings were conducted in accordance with our previous report [10]. The speech recordings were conducted in a quiet, noise-free private room, where a voice recorder, an external microphone, and a PC monitor were set up. The recording equipment used was a TASCAM DR-05 Linear PCM Recorder, with the recording format set to pulse code modulation (PCM) at 44.100 Hz, 16-bit, and mono. The speech tasks included three repetitions of sustained vowels /ɯː, oː, aː, eː, iː/; three repetitions of /apa/, /ata/, and /aka/; and three repetitions of naming, reading aloud, and repetition tasks using two- to five-mora words. The speech tasks were conducted in the following order: repetition, reading aloud, and naming. A total of 10 words were selected, consisting of two-mora words (/basɯ/ (bus) and /sɯzɯ/ (bell)); three-mora words (/zɯboɴ/ (pants), /tsɯkɯe/ (desk), and /nezɯmi/ (mouse)); four-mora words (/kɯtsɯɕita/ (socks), /ɸɯːseɴ/ (balloon), and /naɡaɡɯtsɯ/ (rubber boots)); and five-mora words (/saɴɾiɴɕa/ (tricycle) and /ɾaɴdoseɾɯ/ (school bag)). The selection of words was based on the report by Sugishita et al. [12] and the NTT Japanese database, which provides information on word frequency and familiarity. Sugishita et al. [12] conducted a phonetic analysis of 85 words in individuals with AOS and identified high error rates for consonants /ts/, /dz/, /s/, /g/, and /ɕ/. In the present study, words containing these consonants were included. Additionally, to minimize the cognitive processing load, high-frequency words, as reported by Amano et al. [13], and highly familiar words, as reported by Amano et al. [14], were prioritized.

The acoustic analysis software used in this study was Praat (version 6.4.24). For vowel analysis, the first and second formant frequencies (F1, F2) of the recorded vowels /ɯː oː aː eː iː/ were measured using Praat, specifically focusing on the vowels /aː/, /iː/, and /ɯː/. The tVSA was then calculated based on these values [15]. In this study, the tVSA was used to assess the range of tongue movement in order to differentiate AOS in nfvPPA from dysarthria.

The tVSA and the vowel articulation index (VAI) have been proposed as indicators of vowel centralization, with VAI being particularly effective in minimizing speaker variability and accurately capturing vowel centralization [16]. While the tVSA has the disadvantage of being sensitive to speaker variability, it was chosen in this study due to its extensive use in previous studies, facilitating comparisons with existing research. The tVSA was calculated by plotting F1 and F2 values and applying the following formula:

\(\text{tVSA} = 0.5 \times \Big( ([\text{ɯ}]F_{2} + [\text{i}]F_{2}) \times ([\text{ɯ}]F_{1} - [\text{i}]F_{1}) - ([\text{a}]F_{2} + [\text{ɯ}]F_{2}) \times ([\text{a}]F_{1} - [\text{ɯ}]F_{1}) - ([\text{a}]F_{2} + [\text{i}]F_{2}) \times ([\text{a}]F_{1} - [\text{i}]F_{1}) \Big)\)

The mean and standard deviation (SD) of the three tVSA measurements were calculated.

For VOT analysis, the recorded speech samples of /apa/, /ata/, and /aka/ (each repeated three times) were displayed as waveforms and spectrograms in Praat. To enhance the visualization of plosive energy, the frequency display range was adjusted from the default upper limit of 5,000-10,000 Hz. The plosive sound was identified by listening to the audio while confirming the corresponding spectrogram. The starting point was defined as the onset of energy, and the endpoint was marked at the first peak of the periodic voice waveform (vocal fold vibration). The time interval between the starting and ending points was defined as the VOT. The method for setting the endpoint was based on previous reports [17]. Additionally, the mean VOT and the coefficient of variation (CoV) were calculated to evaluate variability [18].

Speech duration was measured by visually analyzing the waveform and spectrogram of 10 spoken words across naming, reading, and repetition tasks (each repeated three times). The start and end points were determined by both visual inspection and audio listening, and the total speech duration required for word production was recorded. The measurements of tVSA, VOT, and speech duration were conducted by a speech-language pathologist with 18 years of experience in speech assessment, who determined the start and end points and measured the required speech duration. The results are shown in Table 1. Compared to values reported in previous studies of healthy individuals [10], the tVSA in the present case was slightly smaller than that reported for healthy individuals (310,190 ± 37,924 Hz² [10]); however, it remained within a comparable range, indicating that vowel articulation was relatively well preserved. In contrast, the mean values for VOT were elevated across all target syllables. Reference data from healthy individuals show the following VOT means and CoVs: /apa/ (mean: 12.0 ± 2.4 ms, CoV: 0.254 ± 0.054), /ata/ (mean: 16.1 ± 4.8 ms, CoV: 0.163 ± 0.099), and /aka/ (mean: 19.6 ± 5.1 ms, CoV: 0.168 ± 0.035) [10]. Furthermore, speech duration was markedly prolonged in all three tasks - repetition, reading aloud, and naming - relative to normative values (repetition: 0.69 ± 0.08 s; reading aloud: 0.70 ± 0.12 s; naming: 0.70 ± 0.14 s) [10]. Among these, the most pronounced prolongation was observed in the reading aloud task, whereas the naming task exhibited the shortest duration.

**Table 1:** Acoustic features in nfvPPA tVSA: Triangular vowel space area, calculated based on the formant frequencies of corner vowels and expressed in Hz²; VOT: Voice onset time for /apa/, /ata/, and /aka/, measured in seconds; CoV: Coefficient of variation (standard deviation divided by mean), indicating temporal variability; Mean: Average of three repeated measurements for each condition; SD: Standard deviation; nfvPPA: non-fluent/agrammatic variant primary progressive aphasia

tVSA （Hz²)		VOT						Speech duration					
		/apa/ (s)		/ata/ (s)		/aka/ (s)		Repetition (s)		Reading (s)		Naming (s)	
Mean	SD	Mean	CoV	Mean	CoV	Mean	CoV	Mean	SD	Mean	SD	Mean	SD
233421	34806	15.5	0.167	18.3	0.074	24.9	0.198	1.09	0.06	1.19	0.05	1.01	0.06

## Discussion

Among the three recognized variants of PPA, distinguishing the nfvPPA from the lvPPA remains particularly challenging due to the diagnostic ambiguity of AOS, which is included in the classification criteria [8]. The potential utility of acoustic analysis in supporting the diagnosis of nfvPPA in the Japanese language was examined in this case.

Acoustic analysis using tVSA and VOT

In this case, although the patient presented with a shortened lingual frenulum, tongue mobility appeared to be comparable to that of healthy individuals reported in previous studies. Furthermore, the results of the acoustic analysis did not indicate any signs of dysarthria. The mean values of VOT for the syllables /apa/, /ata/, and /aka/ were all elevated. In a previous study, a single case of PPAOS was asked to produce three repetitions each of /apa/, /ata/, and /aka/, and the VOT and its CoV were analyzed. The variability of VOT for the syllable /aka/ has been reported to be significantly greater [10].

In the clinical diagnosis of AOS, perceptual features such as sound distortions and segmentations are commonly used. However, these features are subject to considerable inter-rater variability and may be difficult to distinguish when dysarthria is also present. In contrast, objective acoustic measures such as VOT and CoV provide reliable, quantifiable indices of articulatory timing. These measures are useful not only for supporting diagnosis but also for monitoring longitudinal changes and evaluating treatment outcomes.

In particular, the plosive /k/ tends to have a longer burst release phase due to its articulatory characteristics [19], which makes the VOT more prone to prolongation. In cases of impaired speech motor planning, difficulty in coordinating the release of airflow with vocal fold vibration may lead to further VOT prolongation and increased variability.

Speech duration patterns and the influence of task order

In the present case, speech duration was prolonged across all three tasks - repetition, reading aloud, and naming - compared to values previously reported for healthy individuals [10]. Prior studies have also reported that individuals with nfvPPA exhibit increased relative vowel duration during multisyllabic word production, suggesting that motor planning deficits contribute to delayed speech output. In our previous report involving a PPAOS case, where the tasks were presented in the order of “naming → reading aloud → repetition,” the longest duration was observed during the naming task, and the shortest during repetition [10]. In contrast, in the present case, the tasks were administered in the fixed order of “repetition → reading aloud → naming,” and naming showed the shortest duration. This difference suggests the potential influence of task order or learning effects. Such modality-dependent differences in speech duration were not observed in healthy participants in our prior study [10].

These findings may reflect a heightened sensitivity to speech motor learning mechanisms in individuals with AOS. Aichert et al. [20] reported that syllable-level training is more effective than phoneme-level training for individuals with AOS. Although Japanese is a mora-timed language, the current study did not conduct a detailed analysis based on mora units. However, the observed facilitation effect in this case, possibly resulting from repeated exposure to consistent syllable structures, supports the general notion that speech motor learning is more effective when practiced using larger planning units - such as syllables - regardless of language type.

Limitations

This study is based on a single-case design, which limits the generalizability of the findings. Additionally, the order of speech tasks (repetition → reading aloud → naming) was fixed and not counterbalanced, raising the possibility of order or learning effects. Future studies should address this limitation by implementing a counterbalanced task design to control for potential sequence effects.

## Conclusions

In this single-case study, we analyzed the speech of a Japanese patient with nfvPPA. The patient exhibited prolonged VOT and speech duration. In contrast, vowel articulation, as measured by the tVSA, was relatively preserved. Additionally, the shorter speech duration observed in the naming task compared to repetition and reading tasks may reflect task order or learning effects. These findings suggest that VOT and speech duration may serve as useful objective acoustic markers of AOS in Japanese speakers, providing valuable insights for diagnosis and treatment in languages with prosodic features different from English.
